# Prevalence and Determinants of Chronic Malnutrition among Preschool Children: A Cross-sectional Study in Dhaka City, Bangladesh

**DOI:** 10.3329/jhpn.v29i5.8903

**Published:** 2011-10

**Authors:** Aklima Jesmin, Shelby Suzanne Yamamoto, Ahmad Azam Malik, Md. Aminul Haque

**Affiliations:** ^1^Medicine Sans Frontires Belgium, Dhaka, Bangladesh; ^2^Institute of Public Health, Heidelberg University, Heidelberg, Germany

**Keywords:** Child nutritional disorders, Child nutritional status, Cross-sectional studies, Stunting, Bangladesh

## Abstract

Chronic malnutrition is one of the major causes of morbidity and mortality among preschool children and the future productivity of nations. To understand the prevalence of chronic malnutrition and to identify the factors affecting height-for-age z-score (HAZ) among preschool children, a cross-sectional study was conducted among 380 randomly-selected children aged less than five years in Dhaka city, Bangladesh. Results of analysis of this study data revealed that the prevalence of stunting among preschool children in Dhaka city was 39.5%, with 25% severely stunted and 14% moderately stunted (p<0.001). Results of bivariate analysis revealed that socioeconomic and demographic factors were most significantly associated with the stunting of children. Children were found to be well-nourished if their parents had a tertiary-level education or higher and if the mother held a job and had good knowledge of nutrition. Well-nourishment of the children were also associated with the height of mothers (above 148 cm), good family educational background, normal birthweight, greater frequency of food intake (more than six times/day), and fewer fever episodes in the last six months. Results of multivariate linear regression models showed that height of mothers, birthweight of children, education of fathers, knowledge of mothers on nutrition, and frequency of feeding were the most significant factors that had an independent and direct influence on the stunting of children. To achieve the Millennium Development Goal target of 34% malnutrition prevalence by 2015, it is important to have specific government intervention to focus on the causes that directly influence the stunting of children.

## INTRODUCTION

Malnutrition continues to be a growing problem in most developing countries (1). Poor nutrition during childhood is one important factor impeding the physical and mental development of children, which ultimately propagates the vicious cycle of intergenerational malnutrition. The issue of child malnutrition is critical because its effects are not limited to the boundary of childhood but rather persist into adulthood. It silently destroys the future productivity of nations. Malnutrition increases the economic burden of a society because it leads to increased risk of death from infectious diseases (2), more severe infections, and higher case fatalities (3), creating an additional psychosocial burden. In developing countries, it is estimated that 29% of children aged less than five years (under-five children) are stunted [<-2 standard deviation (SD) height-for-age] or chronically undernourished. Although stunting has declined from 47% in 1980 to 29% in 1995, prevalences are still extremely high, especially in South Central Asia, which alone accounts for about half of the global problem (4). Studying stunting is important because it reflects the cumulative effects of socioeconomic, health and nutritional problems. Stunting is also a predictor of risk because it reflects the overall level of development characterized by poverty, low socioeconomic status, and the prevalence of chronic diseases. It is an important public-health problem in Bangladesh (5).

The prevalence of malnutrition is very high in Bangladesh and is one of the leading causes of morbidity and mortality in children (6,7). Data of the Bangladesh Health and Demographic Survey (BDHS) 2007 show that the prevalence of stunted children is 43.2%, a reduction from 51% in 2004 (8). However, this decline still falls short of the Millennium Development Goal (MDG) 2015 target 34% malnutrition prevalence (9). Currently, 36% of children are born in Bangladesh with low birthweight (4), and 19-27% are severely (10) to moderately stunted (11-13). More than 80% of young infants and 40% of pregnant women are anaemic. Around 400,000 under-five children have severe acute malnutrition, the most severe form of protein-energy malnutrition characterized by a weight-for-height z-score of <-3 or bilateral pedal oedema (14).

Child malnutrition is affected by several determinants, such as intrauterine growth retardation, lack of exclusive breastfeeding, inappropriate complementary feeding, repeated attacks of infectious illnesses, food scarcity, and micronutrient deficiencies (14). The socioeconomic and demographic factors are, importantly, associated with severe and moderate stunting (15). These determinants also vary according to the seasons and spatial distributions of households in the country. The issue of malnutrition is very complex and influenced by multidimensional factors, which have not yet been explored. The present study aims to assess the prevalence of and factors associated with chronic malnutrition among preschool children in the urban setting of Dhaka city, Bangladesh.

## MATERIALS AND METHODS

A cross-sectional study was conducted over a six-month period during 1 December 2007–20 May 2008 in randomly-selected wards (number 45 and 63), the smallest administrative unit of the Dhaka City Corporation, which consists of 90 wards. In total, 380 of 1,265 families were randomly selected that had at least one child aged 0-59 months. Mothers of the selected children were interviewed to obtain information on their socioeconomic, demographic and health and nutrition-related issues through pretested semi-structured questionnaires. The majority of the variables were constructed on an interval scale. Before analysis, the variables were transformed into categorical variables. Anthropometric data were obtained in the same manner to minimize the potential sampling errors. Weight of the child was measured in gramme, and height was measured in centimetre. For children aged less than two years, weight was calculated by subtracting the weight of the mother from the combined weight of the mother-child pair, and recumbent length was measured with a locally-made wooden stadio-meter. Age and the birthweights of children were recorded either from the mother or from the child's delivery discharge paper.

The prevalence of stunting was calculated according to the child growth standard of the World Health Organization (WHO) (16,17). The Anthro 2005 software of the WHO was used for calculating the z-score. This study used the following malnutrition cut-off points recommended by the WHO: (a) severely stunted=z-score <-3 SD; (b) moderately stunted=z-score (-3 to <-2 SD); (c) normal=z-score (-2 to <+1 SD); (d) well-nourished=z-score (+1 to <+2 SD); and (e) obese=z-score (above +2 SD) (18-20).

The independent variables measured in the study included education, height, and nutritional knowledge of mother, education of father, amount of time father spends at home on childcare, monthly family income, birthweight, and frequency of complementary feeding of the child. The variable-economic status—was calculated using assets or a wealth score, rather than income or consumption from the household survey questionnaire of the BDHS. Surveys included questions concerning the household's ownership of 21 of consumer items ranging from a fan to a television and car; dwelling characteristics, such as type of toilet facilities used; and other characteristics relating to wealth. The presence of each item in each household was given a score of 1 or 0 for each consumer item, depending on ownership and summed. Households were sorted into one of five economic score classes as follows:1-5=poor economic class; 6-10=lowermiddle class; 11-15=upper-middle class; and 16-21=high-economic class (Cronbach's alpha coefficient α =0.86) (8).

The variable—family education—is an index of the combined level of education of the respondent, respondent's parents, in-laws, and husband. The total schooling years for all persons in the household were summed and a household mean was calculated. Household means were then coded as follows: very low=0-3; low=4-6; medium=7-10; high=11-14; and very high=15-17. A nutrition knowledge score was calculated based on the mother's responses to the health and nutrition-related items of the questionnaire (Cronbach's α =0.81). The knowledge scores of all mothers were then divided into groups as follows: low=3-6; moderate=7-11; and high=12-16.

To observe the significant variations in the nutritional status of the children among different group means, one-way analysis of variance (ANOVA) using the Scheffe-test and independent sample Student's *t*-tests were applied. Stepwise multiple linear regression analysis was used for modelling the effects of the independent variables on stunting in children. Those variables which were significant at the α =0.05 level were included in the final regression model. All analyses were completed using the SPSS software (version 15.0).

### Ethical approval

The research evaluation committee of the Department of Population Sciences, University of Dhaka, approved the study. The respondents were informed about the study before interview, and their written informed consents were recorded.

## RESULTS

The characteristics of the study children are summarized in Table 1. Their socioeconomic profiles showed that more than 53% of mothers (n=248) had no tertiary education (below graduation), and more than 82% were housewives. However, the nutrition knowledge of 67% of mothers was quite high.

Fathers were more educated than mothers. About 54% of fathers (n=204) had at least 15 years of schooling whereas only 37% of the mothers had the same level of education. The majority (51.6%) of the fathers spent more than two hours at home each day with their children. Family education of 57% of the respondents was recorded as below the medium level, with only 12.1% in the highest category. One quarter of the families had an income of up to Tk 10,000 (US$ 1=Tk 68.5 in 2008). More than 30% of the mothers had a height below 149 cm. More than 20% of the children had low birthweights (below 2,500 g). Around 26% of the mothers gave complementary food (food other than breastmilk) less than four times per day.

Table 1 also presents the distribution of the height-for-age z-scores (HAZ) of the study children according to their socioeconomic, demographic and feeding practices. Results of bivariate analysis showed that the mean HAZ was higher for children of mothers with a postgraduate degree (-0.6316). The primary (-2.256), secondary (-1.854) and higher secondary (-2.331) education groups significantly differed from the postgraduation group. The mean HAZ also showed that the nutritional status of children of working mothers was better compared to that of children of mothers who were housewives. Children of mothers who had good knowledge of nutrition also had significantly better nutritional status than children of mothers with poor nutritional knowledge. The HAZ also significantly varied with education of fathers, with the highest scores from the children of fathers who had postgraduate degrees. The mean HAZ was significantly higher in households where fathers spent more than two hours each day at home on childcare. There was also a significant difference in the mean HAZ between families with very low overall family education and very high family education levels (p=0.028). Additionally, the monthly family income was positively associated with the nutritional status of children, with a higher mean HAZ associated with the monthly family incomes of above Tk 10,000. Height of the mothers was positively associated with the nutritional status of children. A significant difference (p=0.001) was also observed between the height of mothers and the mean z-score of the child. Birthweight was positively associated with nutritional status. Low-birthweight infants were more likely to develop into stunted (-2.396) children than children of normal birthweight (-1.589) and above normal birthweight (-0.849) (21).

Among factors relating to infant feeding, the frequency of complementary feeding was positively associated with the HAZ. In this study, the number of fever episodes in the last six months was negatively associated with the HAZ. There was a significant difference in the mean HAZ among children who did not suffer from fever and those who had between one and three fever episodes in the past six months. Children who suffered from fever for more than six times in the past six months were more likely to be severely stunted (-4.194)

### Prevalence of nutrition

The prevalence of stunted children in the two wards is showed in Table 2. More than 43% of the children were suffering from malnutrition. Of these malnourished children, 25% and 14% were severely and moderately stunted respectively. The prevalence of stunting among preschool children in Dhaka city was significant (p<0.001).

The above model explained 49% of the total variance (Table 3). Height of mothers, education of fathers, birthweight of children, knowledge of mothers on nutrition, and frequency of complementary feeding were the most significant factors for explaining the variance in the stunting of under-five children.

## DISCUSSION

The study was conducted in a rapidly-growing and the most populated city of the country. The respondents, despite their use of available urban facilities, such as electricity, access to media, and sanitation, also suffered from air and sound pollution, insufficient water supply, and electricity shortages. Compared to the overall literacy rate of Bangladesh, the educational qualification of parents in this study was quite high. Despite a fair level of nutritional knowledge of the mothers, the prevalence (43%) of malnutrition among the preschool children was very high. The multivariate results revealed that the practical knowledge of mothers about nutrition was more important for the nutritional status of children than the formal education of mothers. Improved knowledge of the mothers on nutrition significantly reduced the risk of stunting. Conversely, unlike the education of mothers, the education of fathers independently influenced the nutritional status of children. The results of multiple linear regression showed that a one-year increase in schooling of the father can reduce 11% of stunting in children. Apart from stunting, another finding of the study was the emergence of overweight and obesity which are also important public-health concern in the study area.

**Table 1.** Socioeconomic, demographic and feeding practice-related variables of study children and their distribution by their z-score of height-for-age

Characteristics and categories	Children		HAZ	
	%	No.	Mean	p value
Socioeconomic Factors				
Education of mothers ***				
Primary	17.6	67	-2.256	0.001
Secondary	17.9	117	-1.854	0.011
Higher secondary	18.2	64	-2.331	0.001
Graduation	18.4	62	-1.331	0.519
Postgraduation †	18.7	70	-.632	
Work status of mothers ***			
Housewife *	82.1	312	-1.875	0.001
Working mother †	17.9	68	-.865	
Knowledge of mothers on nutrition ***,$				
Low	2.4	9	-4.002	0.002
Moderate	30.3	115	-2.338	<0.001
High †	67.4	256	-1.325	
Education of fathers ***,$				
Primary	14.7	56	-2.273	0.010
Secondary	22.4	85	-2.251	0.002
Higher secondary	9.2	35	-2.061	0.153
Graduation	20.0	76	-1.726	0.226
Postgraduation †	33.7	128	-.953	
Father's spending time (hours) at home ***				
<1	9.5	36	-2.293	0.036
1-2	38.9	148	-2.160	0.001
>2 †	51.6	196	-1.234	
Education of family **				
Very low *	25.3	96	-2.183	0.028
Low	32.6	124	-1.962	0.85
Medium	25.5	97	-1.385	0.770
High †	12.1	46	-.839			
Socioeconomic class *				
Low	11.1	42	-2.388	0.141
Lower-middle class	32.1	122	-1.934	0.396
Upper-middle class	40.5	154	-1.466	0.980
Upper †	16.3	62		
Monthly family income (Tk) ***				
<10,001 †	25	95	-2.368	
10,001-20,000 *	31.8	121	-1.406	0.009
>20,000 *	43.2	164	-1.517	0.015
Demographic factors				
Birthweight of children (g) ***,$				
Low birthweight (<2,500 g) †	20.5	78	-2.396	
Normal weight	67.6	257	-1.589	0.019
Above normal weight *	11.8	45	-.849	0.001
Height (cm) of mothers ***,$				
≤148	30.3	115	-2.841	<0.001
>148	69.7	265	-1.157	
Infant-feeding practice-related factor				
Frequency (times) of complementary feeding ***,$				
<4	25.8	98	-2.345	0.001
4-6	70.5	268	-1.375	<0.001
Sickness-related factors				
No. (times) of fever episodes in the past 6 months *				
No fever	38.9	150	-1.522	0.022
1-3	48.9	186	-1.654	0.033
4-6	9.7	37	-1.838	0.086
>6	1.8	7	-4.197	

*** p<0.001,

** p<0.01,

* p<0.05;

† Reference category to compare the variance among the group means;

$ Variable was selected for multiple linear regression model;

US$ 1=Tk 69.5 (in 2008);

HAZ=Height-for-age z-score;

SD=Standard deviation

Stunting is a predictor of risk as it reflects the overall level of development characterized by poverty, low socioeconomic status, and the prevalence of chronic diseases (15). Nutritional status is related to physical, mental, social and intellectual growth, beginning with foetal development, infancy, and childhood and extending to adolescence and adulthood. Among the demographic factors, birthweight of a child is also a significant factor affecting the risk of stunting as has been found in another study (15).

**Table 2.** Prevalence of stunted children among study subjects

Nutritional status	Frequency	%	p value
Severely stunted	96	25.3	
Moderately stunted	54	14.2	<0.001
Normal	191	50.3	
Well-nourished	24	6.3	
Obese	15	3.9	

**Table 3.** Multiple stepwise regression model of significant variables relating to stunting of study children

Determinant	b	β	R ^2^ change	p value
Height of mother	0.087	0.349	0.158	0.000
Education of father	0.049	0.113	0.043	0.031
Birthweight of child	0.408	0.130	0.020	0.007
Knowledge of mother on nutrition	0.096	0.109	0.011	0.036
Frequency of complementary feeding	0.160	0.097	0.009	0.041

Multiple R=0.490;

R ^2^=0.240;

Adjusted R ^2^=0.229;

Standard error of estimate=1.97;

b=Regression coefficients;

β =Beta-coefficients

### Limitation

A limitation of the present study was that, while conducting the interviews, we had to depend on the information provided by mothers. Information could, thus, have been subject to recall bias. However, we were careful in recording the information they provided and in the interpretation of the results of the study.

### Conclusions

Within the context of the socioeconomic, demographic, educational and spatial factors, the high level of stunting is very much unexpected. People come to the city for better job opportunities, additional income, quality education, improved health, and a healthier future. However, the high level of stunting reflects the drawbacks and problems associated with urban life. To reduce the rates of child mortality and morbidity and to achieve the MDG targets, necessary measures are needed to inform urban families about the high prevalence of malnutrition among their children and to reduce the existing level of stunting.

## ACKNOWLEDGEMENTS

The authors express their gratitude to the United Nations Population Fund and the Department of Population Sciences, University of Dhaka, for financing the study. They are also grateful to all the families who contributed directly and indirectly to the study.
